# Prediction of all-cause and cardiovascular mortality using ankle-brachial index and brachial-ankle pulse wave velocity in patients with type 2 diabetes

**DOI:** 10.1038/s41598-022-15346-9

**Published:** 2022-06-30

**Authors:** Cheng-Chieh Lin, Chia-Ing Li, Chiu-Shong Liu, Chih-Hsueh Lin, Shing-Yu Yang, Tsai-Chung Li

**Affiliations:** 1 School of Medicine, College of Medicine, China Medical University, Taichung, Taiwan R.O.C.; 2 Department of Medical Research, China Medical University Hospital, Taichung, Taiwan R.O.C.; 3 Department of Family Medicine, China Medical University Hospital, Taichung, Taiwan R.O.C.; 4 Department of Public Health, College of Public Health, China Medical University, No. 100, Sec. 1, Jingmao Rd., Beitun Dist., Taichung City, 406040 Taiwan R.O.C.; 5 Department of Healthcare Administration, College of Medical and Health Science, Asia University, Taichung, Taiwan R.O.C.

**Keywords:** Biomarkers, Endocrinology, Risk factors

## Abstract

Ankle-brachial index (ABI) and brachial-ankle pulse wave velocity (baPWV) are used as non-invasive indicators for detecting atherosclerosis and arterial stiffness, two well-known predictors of mortality in patients with type 2 diabetes mellitus (T2DM). ABI and baPWV have independent associations with mortality; however, their joint and interactive effects on mortality have not been assessed in patients with T2DM. This work aims to evaluate the independent, joint, and interactive associations of ABI and baPWV with all-cause and expanded cardiovascular disease (CVD) mortality in patients with T2DM. This observational study included 2160 patients with T2DM enlisted in the Diabetes Care Management Program database of China Medical University Hospital from 2001 to 2016 and then followed their death status until August 2021. Cox proportional hazard models were used to evaluate the independent, joint, and interactive effects of ABI and baPWV on the risk of all-cause and expanded CVD mortality. A total of 474 patient deaths occurred after a mean follow-up of 8.4 years, and 268 of which were attributed to cardiovascular events. Abnormal ABI (≤ 0.9) and highest baPWV quartile were independently associated with increased risks of all-cause [ABI: hazard ratio (HR) 1.67, 95% confidence interval (CI) 1.30–2.11; baPWV: 1.63, 1.16–2.27] and expanded CVD mortality (ABI: 2.21, 1.62–3.02; baPWV: 1.75, 1.09–2.83). The combination of abnormal ABI (≤ 0.9) and highest baPWV quartile was associated with a significantly higher risk of all-cause (4.51, 2.50–8.11) and expanded CVD mortality (9.74, 4.21–22.51) compared with that of the combination of normal ABI and lowest baPWV quartile. Significant interactions were observed between ABI and baPWV in relation to all-cause and expand CVD mortality (both *p* for interaction < 0.001). Through their independent, joint, and interactive effects, ABI and baPWV are significant parameters that can improve the prediction of all-cause and expanded CVD mortality in patients with T2DM and help identify high-risk patients who may benefit from diabetes care.

## Introduction

Diabetes mellitus (DM) is one of the world’s fastest-growing chronic diseases that has affected approximately 463 million people (20–79 years old) in 2019 and has become a heavy health burden because of its high global prevalence^1^. People with DM are prone to chronic complications that are related to macrovascular or microvascular damage and could result in cerebrovascular and cardiovascular events and premature death^2^. Peripheral artery disease (PAD) is the atherosclerosis of lower extremities, such as legs, ankles, or feet, and is also a common complication of type 2 DM that affects around 20–30% of individuals with diabetes^3^. A meta‐analysis showed that diabetes is associated with a high risk of increased morbidity and mortality in PAD^3^. Individuals with DM and PAD are grouped as a high-risk population likely to die from a cardiovascular event. Hence, PAD is recognized as an important predictor for the risk classification of patients with DM.

Ankle–brachial index (ABI) and pulse wave velocity (PWV) are indicators of atherosclerosis and arterial stiffness, respectively^4^. ABI is a simple tool for identifying PAD in clinical practice and is also recommended by the American Heart Association/American College of Cardiology guidelines on the management of patients with lower extremity PAD^5^. For PAD identification, the performance measure of ABI ≤ 0.90 has a sensitivity of 75% (95% CI 71–79%) and a specificity of 86% (95% CI 83–90%), indicating a high level of accuracy^6,7^. Arterial stiffness is one of the earliest stages of atherosclerosis^8^ and may predict cardiovascular morbidity and mortality, especially in individuals with diabetes^9^. Brachial–ankle pulse wave velocity (baPWV) is calculated as the distance between the brachial and tibial arteries divided by the pulse wave travel time between these two arteries and serves as a marker of atherosclerotic vascular damage^10^. baPWV shows 73% sensitivity and 75% specificity at 1635 cm/s with area under receiver operating characteristic curve (AUC) of 0.76 in detecting multiple coronary artery occlusive disease for patients with diabetes^11^ and 60.1% sensitivity and 70.8% specificity at 1874 cm/s with an AUC value of 0.639 in predicting coronary artery disease for elderly patients with chest pain^12^.

A previous comprehensive review indicated that the ABI test is an effective screening tool for preliminary PAD diagnosis and would aid physicians in making a medical decision^6,7^; however, its ability to diagnose PAD in patients with diabetes is limited because its sensitivity is lower for patients with DM than for those without DM^6,7,13^. Diagnostic accuracy could be increased using additional methods, such as ABI in combination with baPWV^6,13,14^. Similar to ABI, baPWV could act as a screening and diagnosis factor for PAD and might even be a better independent predictor than ABI^14^. In addition, the joint and interactive associations of ABI and baPWV with mortality have never been explored. The co-existence of other diseases may modify the effect of the exposure of interest on an outcome^15,16^. To help clarify the joint associations of ABI and baPWV with mortality, this study evaluated whether the combination of ABI and baPWV would have better predictive ability for future all-cause and cardiovascular mortality in among patients with type 2 DM (T2DM) than ABI or baPWV alone. Furthermore, we assessed the interaction of ABI and baPWV, i.e., whether the effect of ABI (or baPWV) on mortality differs depending on the level of baPWV (or ABI).

## Methods

### Study subjects

This retrospective cohort study was conducted among patients with T2DM who were enrolled in the Diabetes Care Management Program (DCMP) of Chinese Medical University Hospital (CMUH) in Taiwan, a case management program established by the National Health Insurance Administration in 2001. The goal of DCMP is to enhance diabetes care quality through intensive monitoring and continuous care to decrease diabetes-related complications. Enrollees are patients with a diagnosis of DM (International Classification Disease, Ninth Revision, Clinical Modification, abbreviated as ICD-9-CM; Code of 250). Eligible cases were those enrolled in the registry between November 2001 and April 2016 and were followed up to August 2021 or until death. This cohort was open or dynamic because of each subject entering the study at different time points with unequal follow-up intervals. Index date was defined as the date of entry into the DCMP. We excluded patients with type 1 diabetes (ICD-9-CM code 250. × 1/ × 3), gestational diabetes (ICD-9-CM code 648.83), patients aged under 30 years, and those without ABI and baPWV values. A total of 2,390 subjects were eligible at this time (Supplemental Fig. S1). We further excluded those who followed up < 1 year and had missing data of sociodemographic factors, lifestyle behaviors, diabetes-related factor, complications, medication use, and biomarkers. The patients who had < 1 year of follow-up were excluded because they cannot provide data for subsequent mortality status to rule out the possibility of reverse causality. A total of 2,160 participants were finally included for analysis. Details of the procedures used have been reports previously^17–19^. This study was approved by the Research Ethics Committee of China Medical University Hospital (CMUH110-REC1-204), and all methods were performed in accordance with the relevant guidelines and regulations.

### Data source

Data were retrieved from the computerized database of patients with T2DM enrolled in the DCMP of CMUH in Taichung, Taiwan. DCMP requires health care providers to participate in clinical education and training programs for certification to become eligible to voluntarily enroll patients into this program. The health care providers consist of physicians from endocrinology, internal medicine, family medicine, nephrology, cardiology, and other specialties. The continuing education and training programs promote the standardization of clinical practice, such as the assessment and diagnosis of diabetic complications. Coordinated care provided by physician-led multidisciplinary teams includes physicians and their medical care staff working to adhere to established clinical guideline for diabetes care. This database of DCMP provides information on patients with diabetes, including annual self-care education and assessments, eye examinations, and laboratory tests. Laboratory tests include fasting plasma glucose (FPG), hemoglobin A1c (HbA1c), high density lipoprotein-cholesterol (HDL-C), low density lipoprotein-cholesterol (LDL-C), triglyceride (TG), and total cholesterol (TC). The DCMP database also provides data on lifestyle factors, including smoking, alcohol drinking, regular physical activity, and family history of disease. The medications include information about oral hypoglycemic agents, insulin, antihypertensive agents, cholesterol-lowering agents, and cardiovascular agents. In addition to the laboratory and pharmacy data regulated by DCMP for reimbursement, information for education on nutrition, nursing, diet, and weight control behaviors was retrieved.

### Measurements

Upon entering the DCMP, patients underwent a series of medical tests for blood, urine, and body measurements and completed a computerized questionnaire for lifestyle, activity, and medical history administered by a case management nurse to record previous or current disease status. The description of variables is shown below.

#### Socio-demographic factors, lifestyle factors, and diabetes-related variables

The socio-demographic factors include age at baseline, gender and family history of diabetes, hypertension, hyperlipidemia, and obesity. Lifestyle factors of smoking, alcohol drinking, and physical activity were divided into two responses based on participants’ self-reports: yes or no.

#### Drug-related variables and medications

Diabetes-related variables include the duration and treatment of T2DM. Data on the types of diabetes treatment such as oral hypoglycemic agents of metformin, sulfonylurea, thiazolidinedione, meglitinide, and biguanide, and insulin therapy were extracted from the electronic medical records. Information for the use of pharmacologic agents was derived from the DCMP dataset. Drug-related variables include hypertension medications (e.g., calcium channel blockers), hyperlipidemia medications (e.g., statins [HMG-CoA reductase inhibitors]), and cardiovascular medications. All medications were divided into two responses based on electronic medical record: yes or no.

#### Comorbidities

Baseline diabetic acute complications include diabetic ketoacidosis, hyperglycemic hyperosmolar nonketotic coma, and severe hypoglycemia. Chronic complications of hypertension consist of hyperlipidemia, stroke, coronary artery disease, peripheral neuropathy, and nephropathy. The time frame for measuring these comorbidities was over a 1-year period prior to the interview. All diabetic comorbidities were divided into two responses: yes or no.

#### Anthropometric measurements

Weight and height were measured on an autoanthropometer (super-view, HW-666) with the subjects being shoeless and wearing light clothing. Body mass index (BMI) was calculated as weight (kg)/(height)^2^ (m^2^). Blood pressure (BP) was measured three times in the right arm of the patient in a seated position without distraction by using a suitable size cuff and a standard tunnel type electronic sphygmomanometer (OMRON, HBP-9020, Japan). Individuals were asked to rest and sit up straight in a chair next to a table for 5–10 min with their arm resting comfortably at heart level, their back against the chair, legs uncrossed, and their forearm resting on the table with the palm of their hand facing up. Individuals usually had one BP measurement. If an individual had two or more BP measurements in a day, then the average was calculated.

#### Laboratory examination

Blood was drawn from an antecubital vein in the morning after a 12 h overnight fasting and was sent for analysis within 4 h after collection. Biochemical markers such as FPG, HbA1c, HDL, LDL, TC, and TG were analyzed by a biochemical auto-analyzer (Beckman Coulter Synchron system, Lx-20, Fullerton, CA, USA) at the Clinical Laboratory Department of CMUH. FPG level was measured in the blood collected in a NAF TUBE containing 5 mg of sodium fluoride to inhibit glucose metabolism and 4 mg of potassium oxalate to chelate calcium and prevent coagulation. Inter- and intra-assay coefficient of variations (CVs) for FPG were both 4%. HbA1c level was measured by boronate-affinity high-performance liquid chromatography (HPLC) assay (reference range, 4.6% to 6.5%). The inter- and intra-assay CVs for HbA_1c_ were 2.91% for the normal level, 1.79% for the intermediate level, and 1.09% for the high level. TC and TG levels were measured in serum mode. TG levels were determined by an enzymatic colorimetric method, and the inter- and intra-assay CVs were 6.8% and 5%, respectively. HDL and LDL levels were measured by a direct method, and the inter- and intra-assay CVs were both 4.5% for HDL and 4.5% and 3%, respectively, for LDL.

#### ABI and baPWV

ABI and baPWV were measured using pressure cuffs wrapped around the brachium and ankle^20^ and determined using an automatic volume-plethysmographic device PWV/ABI (PWV/ABI; Colin Co., Ltd., Komaki, Japan), which simultaneously records PWV, BP, electrocardiograph, and heart sounds^20,21^. Validation studies using Pearson’s correlation analysis and Bland–Altman plot revealed that this automatic non-invasive device has good validity and reproducibility^20,22,23^ and could be used to screen subclinical vascular pathology^24^, and its reproducibility was previously documented (CV = 8.4% and reproducibility coefficient = 0.98)^20^. The subjects were examined in the supine position after at least 5 min of rest. Electrodes of the electrocardiograph were placed on both wrists, and pneumatic cuffs were placed on the brachium and ankles. The cuffs were connected to a sensor that determines volume pulse from and to an oscillometric pressure sensor that measures the BP. Heart sounds S1 and S2 were detected by a microphone placed on the left edge of the sternum at the fourth intercostal space. The time interval between the wave fronts of the brachial waveform and ankle waveform was determined as the time interval between the brachium and ankle (△T_ba_). The sampling time was 10 s with automatic gain analysis and quality adjustment. The distance between the sampling points of baPWV was calculated automatically according to the subject’s height. The path length from the heart to the brachium (L_b_) was expressed using the following equation: L_b_ = 0.2195 * height of the patient (in cm) − 2.0734. The path length from the heart to the ankle (L_a_) was expressed by the following equation: L_a_ = 0.8129 * height of the patient (in cm) + 12.328. baPWV was computed as baPWV = (L_b_ − L_a_)/△T_ba_ (cm/s). The right brachial-to-right ankle PWV was calculated between the right arm and left ankle, and the right brachial-to-left ankle PWV was measured between the right arm and left ankle. High baPWV indicates severe arteriosclerosis. We selected the higher baPWV between the above values as the representative baPWV for indexing arterial stiffness. ABI was derived from dividing the systolic BP at the arteries near the ankle by the systolic BP in the arms. A low ABI indicates severe atherosclerosis. We selected the lower value between the right and left ABI as the representative ABI for indexing atherosclerosis. An ABI ≤ 0.9 was defined as abnormal^25^.

#### Outcome measures

All-cause and expanded cardiovascular disease (CVD) mortality was determined through annual record linkage with National Death Datasets by using data on personal identification number and date of death provided by the Taiwan Ministry of Health and Welfare. All patients were followed up from the index date up to August, 2021 or until death. The underlying causes of death were coded in accordance with either ICD-9-CM for the cases that occurred from 2006 to 2008 or International Classification of Disease, 10th Revision, Clinical Modification (ICD-10-CM) for the cases that occurred from 2009 to 2021. Expanded CVD mortality was defined as death due to CVD (ICD-9-CM codes 390–459, ICD-10-CM codes I00–I99) plus diabetes (ICD-9-CM code 250, ICD-10-CM codes E10–E14) or plus kidney diseases (ICD-9-CM 580–589; ICD-10-CM N00–N29). We considered the expanded CVD mortality because it was a composite measure of cardiovascular-related mortality, i.e., a derived variable based on multiple items that are cardiovascular-related causes of death^26^. We assumed that persons with diabetes are likely to die from diabetes and other CVDs. Meanwhile, chronic kidney disease is a key risk factor for CVDs. Thus, the deaths due to chronic renal disease are considered as CVD-related death. Under this condition, the expanded CVD mortality has the advantage of increased statistical power to detect the association of interest.

### Statistical analysis

Simple descriptive analyses such as mean, standard deviation, and proportion were employed to analyze data when appropriate. Baseline variables for survivors and non-survivors were assessed by the Chi-Square tests for categorical variables and t tests for continuous variables. The subjects were divided into groups according to normal ABI (ABI > 0.9), abnormal ABI (ABI ≤ 0.9), or baPWV quartiles. AUCs were used to evaluate the diagnostic ability of ABI, baPWV, and their combination. Sensitivity, specificity, positive predictive value (PPV), and negative predictive value (NPV) were calculated to determine the accuracy and predictive ability of the diagnostic tests. Moreover, to assess the incremental discriminatory ability of ABI alone, baPWV alone, and their combination on all-cause and expanded CVD mortality risk stratification, we employed Harrell’s C-index, continuous version net reclassification improvement (NRI), and integrated discrimination improvement (IDI) to quantify the improvement in risk discrimination^27^. Cox proportional hazard models and restricted cubic splines were employed to explore the independent effects of ABI and baPWV on all-cause and expanded CVD mortality. The joint effect of ABI and baPWV on all-cause and expanded CVD mortality were estimated through the derived variable of combined ABI and baPWV subgroups. We also tested the interaction of ABI and baPWV by entering a product term of ABI and baPWV subgroups into the Cox models and testing its significance using the likelihood ratio test. All analyses were performed with SAS version 9.4 (SAS, Cary, NC). All *P* values were two-tailed, and a *P* value < 0.05 was considered statistically significant.

### Ethics approval and consent to participate

This study was approved by the Ethical Review Board of China Medical University Hospital (CMUH110-REC1-204). Informed consent of the study participants was not required because the dataset used in this study consists of de-identified secondary data released for research purposes.

## Results

A total of 2160 diabetic patients aged 30 years or older were included in this study. During the average follow-up of 8.4 years, 474 (22%) of the patients died (8.2% from expanded CVD events). Table 1 describes the baseline characteristics analyzed according to the survival status. Compared with the non-survivors, the survivors were younger and had shorter duration of diabetes, higher levels of BMI, LDL-C, and TC, and less proportions of oral hypoglycemic drug plus insulin use, hypertension, stroke, coronary artery disease, peripheral neuropathy, nephropathy, hypertension medication use, and cardiovascular medication use. The patients were divided into normal ABI (ABI > 0.9, n = 1915) or abnormal ABI (ABI ≤ 0.9, n = 245) and baPWV quartiles. Table 2 presents the baseline characteristics of the patients grouped by ABI and baPWV. Compared with the other patients, those with abnormal ABI or highest baPWV quartile were older, women, and had longer duration of diabetes, higher HbA1c level, and higher proportions of oral hypoglycemic drug plus insulin use, hypertension, stroke, coronary artery disease, peripheral neuropathy, nephropathy, hypertension medication use, and cardiovascular medications use.

**Table 1:** The comparisons of baseline sociodemographic factors, life style behaviors, diabetes-related variables, complications, medication use and biomarkers according to death status in patients with type 2 diabetes (n = 2160).

Variables	Death N (%)		*P* value
	No (*n* = 1686)	Yes (*n* = 474)	
**Sociodemographic factors**			
Sex			0.98
Men	957 (56.76)	270 (56.96)	
Women	729 (43.24)	204 (43.04)	
Age (years)^†^	59.04 ± 10.34	68.65 ± 10.87	< 0.001
**Life style behaviors**			
Smoking	328 (19.45)	97 (20.46)	0.67
Alcohol drinking	193 (11.45)	35 (7.38)	0.01
Exercising	868 (51.48)	250 (52.74)	0.67
BMI (kg/m^2^)^†^	26.08 ± 3.96	25.51 ± 4.10	0.007
**Diabetes-related variables**			
Duration of diabetes (years)^†^	8.65 ± 7.34	12.85 ± 9.05	< 0.001
Types of diabetes treatment			< 0.001
Diet or exercise	77 (4.57)	28 (5.91)	
Oral hypoglycemic drug	1396 (82.8)	330 (69.62)	
Inject insulin	14 (0.83)	8 (1.69)	
Both	199 (11.80)	108 (22.78)	
**Complications**			
Hypertension	557 (33.04)	208 (43.88)	< 0.001
Hyperlipidemia	376 (22.30)	97 (20.46)	0.43
Stroke	43 (2.55)	37 (7.81)	< 0.001
Coronary artery disease	68 (4.03)	68 (14.35)	< 0.001
Severe hypoglycemia	7 (0.42)	4 (0.84)	0.43
Peripheral neuropathy	143 (8.48)	100 (21.10)	< 0.001
Nephropathy	60 (3.56)	65 (13.71)	< 0.001
DKA	5 (0.30)	0 (0.00)	0.59
HHNK	3 (0.18)	3 (0.63)	0.24
**Medication use**			
Hypertension medications	549 (32.56)	192 (40.51)	0.002
Hyperlipidemia medications	212 (12.57)	48 (10.13)	0.17
Cardiovascular medications	220 (13.05)	105 (22.15)	< 0.001
**Biomarkers**^†^			
HbA1c (%)	7.88 ± 1.68	8.21 ± 1.91	< 0.001
FPG (mg/dL)	152.26 ± 49.72	158.81 ± 75.2	0.07
LDL-C (mg/dL)	113.66 ± 34.09	108.67 ± 34.04	0.005
HDL-C (mg/dL)	42.51 ± 10.74	41.32 ± 12.39	0.06
TC (mg/dL)	189.67 ± 40.79	184.91 ± 44.27	0.04
TG (mg/dL)	170.90 ± 201.63	161.51 ± 148.74	0.26

Student’s t-test was used for continuous variables to calculate *P* values.

Chi-square test or Fisher's Exact Test was used for categorical variables to calculate *P* values.

^†^Mean ± SD; BMI: body mass index: DKA: diabetic ketoacidosis; HHNK: hyperglycemic hyperosmolar nonketotic coma; FPG: fasting plasma glucose; LDL-C: low density lipoprotein-cholesterol; HDL-C: high density lipoprotein-cholesterol; TC: total cholesterol; TG: triglyceride.

**Table 2:** The comparisons of baseline sociodemographic factors, life style behaviors, diabetes-related variables, complications, medication use and biomarkers according to ABI and baPWV in patients with type 2 diabetes (n = 2160).

Variables	ABI		*P* value	baPWV (cm/s)				*P* value
	> 0.9 (*n* = 1915)	≤ 0.9 (*n* = 245)		< 1515 (*n* = 540)	1515–1759 (*n* = 538)	1760–2069 (*n* = 543)	≥ 2070 (*n* = 539)	
**Sociodemographic factors**								
Sex			0.02					< 0.001
Men	1105 (57.7)	122 (49.8)		368 (68.15)	327 (60.78)	285 (52.49)	247 (45.83)	
Women	810 (42.3)	123 (50.2)		172 (31.85)	211 (39.22)	258 (47.51)	292 (54.17)	
Age (years)^†^	60.18 ± 10.76	68.72 ± 11.59	< 0.001	52.93 ± 9.70	58.47 ± 9.36	64.07 ± 9.36	69.10 ± 9.25	< 0.001
**Life style behaviors**								
Smoking	363 (18.96)	62 (25.31)	0.02	141 (26.11)	122 (22.68)	98 (18.05)	64 (11.87)	< 0.001
Alcohol drinking	208 (10.86)	20 (8.16)	0.24	71 (13.15)	64 (11.9)	58 (10.68)	35 (6.49)	0.003
Exercising	1013 (52.9)	105 (42.86)	0.004	260 (48.15)	278 (51.67)	289 (53.22)	291 (53.99)	0.23
BMI (kg/m^2^)^†^	25.98 ± 3.96	25.74 ± 4.3	0.38	26.37 ± 4.11	26.06 ± 4.08	25.98 ± 4.01	25.39 ± 3.74	< 0.001
**Diabetes-related variables**								
Duration of diabetes (years)^†^	9.05 ± 7.57	13.62 ± 9.46	< 0.001	4.07 ± 5.94	5.74 ± 6.36	7.10 ± 7.05	8.73 ± 7.96	< 0.001
Types of diabetes treatment			< 0.001					< 0.001
Diet or exercise	89 (4.65)	16 (6.53)		36 (6.67)	20 (3.72)	23 (4.24)	26 (4.82)	
Oral hypoglycemic drug	1556 (81.25)	170 (69.39)		446 (82.59)	452 (84.01)	421 (77.53)	407 (75.51)	
Inject insulin	17 (0.89)	5 (2.04)		6 (1.11)	5 (0.93)	6 (1.1)	5 (0.93)	
Both	253 (13.21)	54 (22.04)		52 (9.63)	61 (11.34)	93 (17.13)	101 (18.74)	
**Complications**								
Hypertension	652 (34.05)	113 (46.12)	< 0.001	108 (20.00)	198 (36.8)	223 (41.07)	236 (43.78)	< 0.001
Hyperlipidemia	416 (21.72)	57 (23.27)	0.64	106 (19.63)	128 (23.79)	120 (22.1)	119 (22.08)	0.43
Stroke	53 (2.77)	27 (11.02)	< 0.001	9 (1.67)	13 (2.42)	26 (4.79)	32 (5.94)	< 0.001
Coronary artery disease	101 (5.27)	35 (14.29)	< 0.001	19 (3.52)	32 (5.95)	35 (6.45)	50 (9.28)	0.002
Severe hypoglycemia	8 (0.42)	3 (1.22)	0.12	1 (0.19)	1 (0.19)	2 (0.37)	7 (1.30)	0.05
Peripheral neuropathy	186 (9.71)	57 (23.27)	< 0.001	33 (6.11)	55 (10.22)	65 (11.97)	90 (16.7)	< 0.001
Nephropathy	95 (4.96)	30 (12.24)	< 0.001	18 (3.33)	20 (3.72)	37 (6.81)	50 (9.28)	< 0.001
DKA	5 (0.26)	0 (0.00)	1.00	2 (0.37)	1 (0.19)	1 (0.18)	1 (0.19)	0.94
HHNK	5 (0.26)	1 (0.41)	0.51	1 (0.19)	0 (0.00)	0 (0.00)	5 (0.93)	0.008
**Medication use**								
Hypertension medications	624 (32.58)	117 (47.76)	< 0.001	121 (22.41)	177 (32.9)	215 (39.59)	228 (42.30)	< 0.001
Hyperlipidemia medications	227 (11.85)	33 (13.47)	0.53	66 (12.22)	67 (12.45)	59 (10.87)	68 (12.62)	0.81
Cardiovascular medications	263 (13.73)	62 (25.31)	< 0.001	57 (10.56)	61 (11.34)	102 (18.78)	105 (19.48)	< 0.001
**Biomarkers**^†^								
HbA1c (%)	7.91 ± 1.74	8.25 ± 1.69	0.004	7.77 ± 1.73	7.90 ± 1.65	8.05 ± 1.73	8.10 ± 1.82	0.008
FPG (mg/dL)	153.29 ± 55.6	156.87 ± 61.97	0.39	148.65 ± 48.53	155.77 ± 58.72	154.38 ± 52.96	155.99 ± 63.9	0.11
LDL-C (mg/dL)	112.35 ± 33.88	114.28 ± 36.08	0.40	113.23 ± 35	111.63 ± 34.18	112.45 ± 32.48	112.95 ± 34.9	0.88
HDL-C (mg/dL)	42.49 ± 11.12	40.41 ± 11.11	0.006	41.59 ± 9.98	41.93 ± 10.76	42.4 ± 12.08	43.1 ± 11.56	0.13
TC (mg/dL)	187.9 ± 41.02	194.3 ± 45.69	0.04	188 ± 39.41	187.53 ± 42.63	187.94 ± 40.33	191.03 ± 43.95	0.49
TG (mg/dL)	166.96 ± 196.97	183.56 ± 138.58	0.10	173.68 ± 187.41	176.85 ± 230.77	160.18 ± 128.46	164.72 ± 204.19	0.45

Student’s t-test or ANOVA was used for continuous variables to calculate *P* values.

Chi-square test or Fisher's Exact Test was used for categorical variables to calculate *P* values.

^†^Mean ± SD; CKD: chronic kidney disease; BMI: body mass index: DKA: diabetic ketoacidosis; HHNK: hyperglycemic hyperosmolar nonketotic coma; FPG: fasting plasma glucose; LDL-C: low density lipoprotein-cholesterol; HDL-C: high density lipoprotein-cholesterol; TC: total cholesterol; TG: triglyceride.

The incidence densities of all-cause mortality were 21.21 and 79.17 per 1000 person-years in patients with normal and abnormal ABI, respectively, and 11.21, 17.51, 27.57, and 54.09, per 1000 person-years in patients with the lst, 2nd, 3rd, and 4th quartiles of baPWV, respectively. Table 3 presents the hazard ratios of all‐cause and expanded CVD mortality for ABI and baPWV. Compared with the individuals with normal ABI or lowest baPWV quartile, those with abnormal ABI and highest baPWV quartile were independently associated with higher risks of all-cause [ABI: hazard ratio (HR) 2.34, 95% confidence interval (CI) 1.88–2.91; baPWV: 1.90, 1.37–2.65] and expanded CVD mortality (ABI: 3.30, 2.47–4.42; baPWV: 2.25, 1.40–3.62) according to the model adjusted for age and sex only. In multivariate analysis, abnormal ABI and highest baPWV quartile were still independently associated with all-cause (ABI: 1.67, 1.30–2.11; baPWV: 1.63, 1.16–2.27) and expanded CVD mortality (ABI: 2.21, 1.62–3.02; baPWV: 1.75, 1.09–2.83).

**Table 3:** Hazard ratios of all-cause mortality and expanded CVD mortality for ABI and baPWV in patients with type 2 diabetes (n = 2160).

	*n*	Case	Person-years	IR	Multivariate model 1		Multivariate model 2		Multivariate model 3	
					HR (95% CI)	*P*	HR (95% CI)	*P*	HR (95% CI)	*P*
**All-cause mortality**										
ABI										
> 0.9	1915	350	16,498.61	21.21	1.00		1.00		1.00	
≤ 0.9	245	124	1566.27	79.17	2.34 (1.88, 2.91)	< 0.001	1.80 (1.43, 2.26)	< 0.001	1.67 (1.32, 2.11)	< 0.001
baPWV (cm/s)										
< 1515	540	55	4907.54	11.21	1.00		1.00		1.00	
1515–1759	538	83	4740.44	17.51	1.11 (0.79, 1.57)	0.55	1.08 (0.76, 1.52)	0.67	1.08 (0.77, 1.53)	0.65
1760–2069	543	124	4497.74	27.57	1.24 (0.89, 1.73)	0.21	1.18 (0.84, 1.64)	0.34	1.15 (0.82, 1.61)	0.41
≥ 2070	539	212	3919.16	54.09	1.90 (1.37, 2.65)	< 0.001	1.70 (1.22, 2.37)	0.002	1.63 (1.16, 2.27)	0.004
*P* for trend					< 0.001		< 0.001		< 0.001	
**Expanded CVD mortality**										
ABI										
> 0.9	1915	161	16,498.61	9.76	1.00		1.00		1.00	
≤ 0.9	245	77	1566.27	49.16	3.30 (2.47, 4.42)	< 0.001	2.50 (1.84, 3.40)	< 0.001	2.21 (1.62, 3.02)	< 0.001
baPWV (cm/s)										
< 1515	540	26	4907.54	5.30	1.00		1.00		1.00	
1515–1759	538	41	4740.44	8.65	1.17 (0.71, 1.92)	0.53	1.14 (0.69, 1.88)	0.60	1.15 (0.70 1.90)	0.59
1760–2069	543	56	4497.74	12.45	1.21 (0.74, 1.96)	0.45	1.10 (0.68, 1.79)	0.69	1.05 (0.65, 1.71)	0.84
≥ 2070	539	115	3919.16	29.34	2.25 (1.40, 3.62)	< 0.001	1.94 (1.21, 3.12)	0.006	1.75 (1.09, 2.83)	0.02
*P* for trend					< 0.001		0.001		0.007	

Multivariate model 1 adjusted for age and sex.

Multivariate model 2 adjusted for smoking, alcohol drinking, exercising, body mass index (BMI), duration of diabetes, types of diabetes treatment, HbA1c, FPG, LDL-C, HDL-C, TG and TC, in addition to the variables in the multivariate model 1.

Multivariate model 3 adjusted for baseline status of hypertension, hyperlipidemia, stroke, coronary artery disease, severe hypoglycemia, peripheral neuropathy, nephropathy, diabetic ketoacidosis (DKA), hyperglycemic hyperosmolar nonketotic coma (HHNK), hypertension medications, hyperlipidemia medications and cardiovascular medications, in addition to the variables in the multivariate model 2.

Table 4 presents the sensitivity, specificity, PPV, and NPV for different cutoff points of ABI and baPWV. The cutoff point of ABI ≤ 0.9 showed 26.16% sensitivity, 92.82% specificity, 50.61% PPV, and 81.72% NPV for all-cause mortality and 32.35% sensitivity, 91.26% specificity, 31.43% PPV, and 91.59% NPV for expanded CVD mortality. Given that the cutoff points of ABI were specified at high values, the values of sensitivity and NPV increased but those of specificity and PPV decreased. On the contrary, the cutoff point of baPWV ≥ 1500 showed 88.61% sensitivity, 27.46% specificity, 25.56% PPV, and 89.56% NPV for all-cause mortality and 89.08% sensitivity, 25.55% specificity, 12.90% PPV, and 94.97% NPV for expanded CVD mortality. Given that the cutoff points of baPWV were specified at high values, the values of sensitivity and NPV decreased but those of specificity and PPV increased.

**Table 4:** Accumulated incidence of all-cause and expanded CVD mortality appearing during the follow-up period according to presence of pathological ABI and baPWV at baseline.

Cutoff point for high risk	All-cause mortality (*n* = 474)					Expanded CVD mortality (*n* = 238)				
	*n* (%)	Sensitivity	Specificity	PPV	NPV	*n* (%)	Sensitivity	Specificity	PPV	NPV
**ABI**										
≤ 0.8	86 (18.14)	18.14	95.73	54.43	80.62	56 (23.53)	23.53	94.69	35.44	90.91
≤ 0.9	124 (26.16)	26.16	92.82	50.61	81.72	77 (32.35)	32.35	91.26	31.43	91.59
≤ 1.0	194 (40.93)	40.93	81.91	38.88	83.14	115 (48.32)	48.32	80.02	23.05	92.59
≤ 1.1	318 (67.09)	67.09	42.29	24.63	82.05	171 (71.38)	71.85	41.73	13.25	92.29
≤ 1.2	445 (93.88)	92.62	7.83	22.03	79.04	226 (94.96)	94.96	8.06	11.34	92.81
**baPWV (cm/s)**										
≥ 1400	445 (93.88)	93.88	15.78	23.86	90.17	223 (93.70)	93.70	14.57	11.96	94.92
≥ 1500	420 (88.61)	88.61	27.46	25.56	89.56	212 (89.08)	89.08	25.55	12.90	94.97
≥ 1600	388 (81.86)	81.86	38.14	27.11	88.20	196 (82.35)	82.35	35.74	13.70	94.24
≥ 1700	358 (75.53)	75.53	49.58	29.64	87.82	180 (75.63)	75.63	46.51	14.90	93.91
≥ 1800	323 (68.14)	68.14	60.02	32.40	87.02	169 (71.01)	71.01	56.92	16.95	94.07
≥ 1900	282 (59.49)	59.49	68.80	34.90	85.80	151 (63.45)	63.45	65.82	18.69	93.57
≥ 2000	242 (51.05)	51.05	75.92	37.46	84.54	127 (53.36)	53.36	73.00	19.66	92.67

PPV, positive predictive value; NPV, negative predictive value.

**Figure 1:**
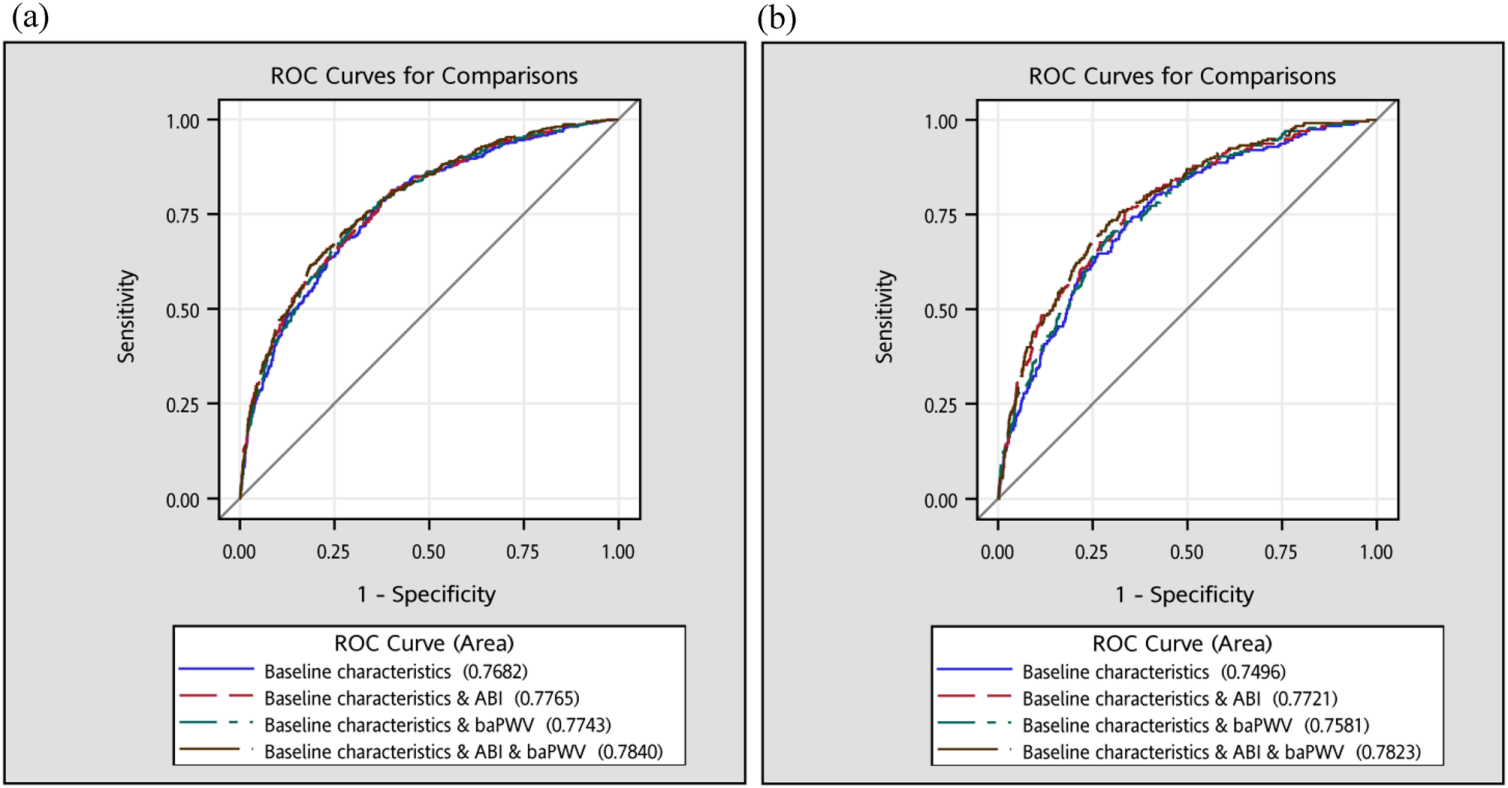
The areas under the receiver operating characteristic curves for (**a**) all-cause mortality, and (**b**) expanded CVD mortality.

The combination of ABI and baPWV had significantly better predictive ability than ABI or baPWV alone for all-cause mortality (AUC of 0.7840 vs. 0.7765, *P* = 0.011; AUC of 0.7840 vs. 0.7743, *P* = 0.004) and expanded CVD mortality (AUC of 0.7823 vs. 0.7721, *P* = 0.035; AUC of 0.7823 vs. 0.7581, *P* < 0.001) (Fig. 1). The addition of ABI alone, baPWV alone, and their combination exhibited significant enhancement on risk stratification compared with that of the model with baseline characteristics for all-cause and expanded CVD mortality (all *P* < 0.05) except ABI alone for continuous version NRI on all-cause and expanded CVD mortality. The maximum differences were observed in ABI and baPWV combination for continuous version NRI of 0.072 (0.033, 0.111) and IDI of 0.026 (0.018, 0.035) on all-cause mortality and for continuous version NRI of 0.133 (0.074, 0.192) and IDI of 0.032 (0.019, 0.044) on expanded CVD mortality (Table 5).

**Table 5:** Incremental discriminatory ability of ABI alone, baPWV alone, and their combination of the prediction of all-cause and expanded CVD mortality.

Models	Harrell’s c-statistic	Continuous version NRI		IDI	
	Estimate (95% CI)	Difference (95% CI)	*P* value	Difference (95% CI)	*P* value
**Prediction of all-cause mortality**					
Model with baseline characteristics^a^	0.760 (0.737, 0.783)	Reference		Reference	
With ABI added	0.767 (0.745, 0.789)	0.022 (−0.009, 0.053)	0.17	0.018 (0.010, 0.025)	< 0.001
With baPWV added	0.766 (0.743, 0.788)	0.055 (0.020, 0.090)	0.002	0.009 (0.004, 0.014)	< 0.001
With ABI and baPWV added	0.773 (0.751, 0.795)	0.072 (0.033, 0.111)	< 0.001	0.026 (0.018, 0.035)	< 0.001
**Prediction of expanded CVD mortality**					
Model with baseline characteristics^a^	0.778 (0.747, 0.810)	Reference		Reference	
With ABI added	0.795 (0.766, 0.825)	0.032 (−0.019, 0.084)	0.22	0.024 (0.013, 0.035)	< 0.001
With baPWV added	0.784 (0.754, 0.815)	0.057 (0.010, 0.104)	0.02	0.009 (0.003, 0.015)	0.006
With ABI and baPWV added	0.802 (0.774, 0.831)	0.133 (0.074, 0.192)	< 0.001	0.032 (0.019, 0.044)	< 0.001

CI, confidence interval; NRI, net reclassification improvement; IDI, integrated discrimination improvement.

^a^Baseline characteristics included age, sex, smoking, alcohol drinking, exercising, duration of diabetes, and types of diabetes treatment.

**Figure 2:**
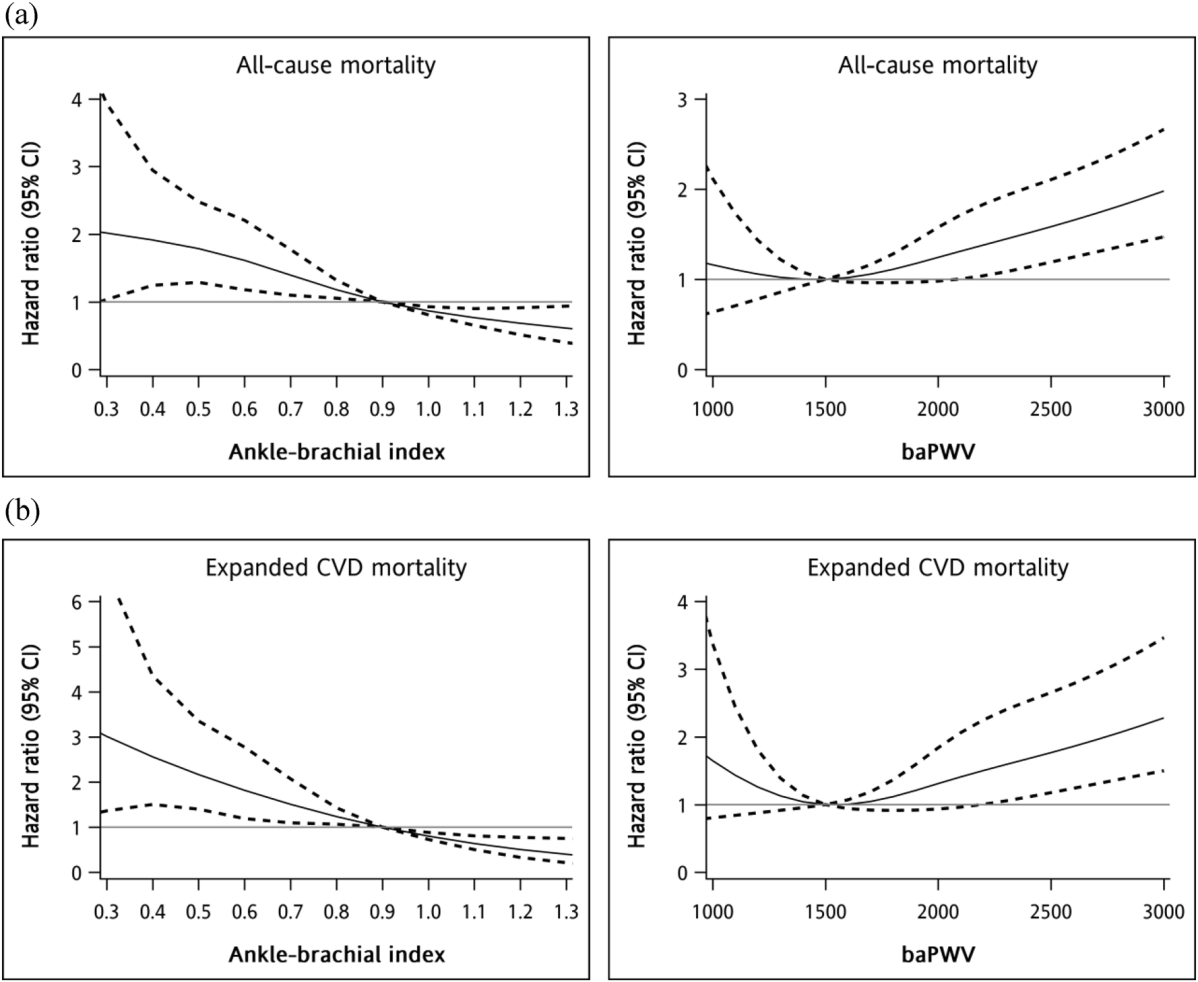
Multivariable cubic spline plots for (**a**) all-cause mortality and (**b**) expanded CVD mortality.

**Figure 3:**
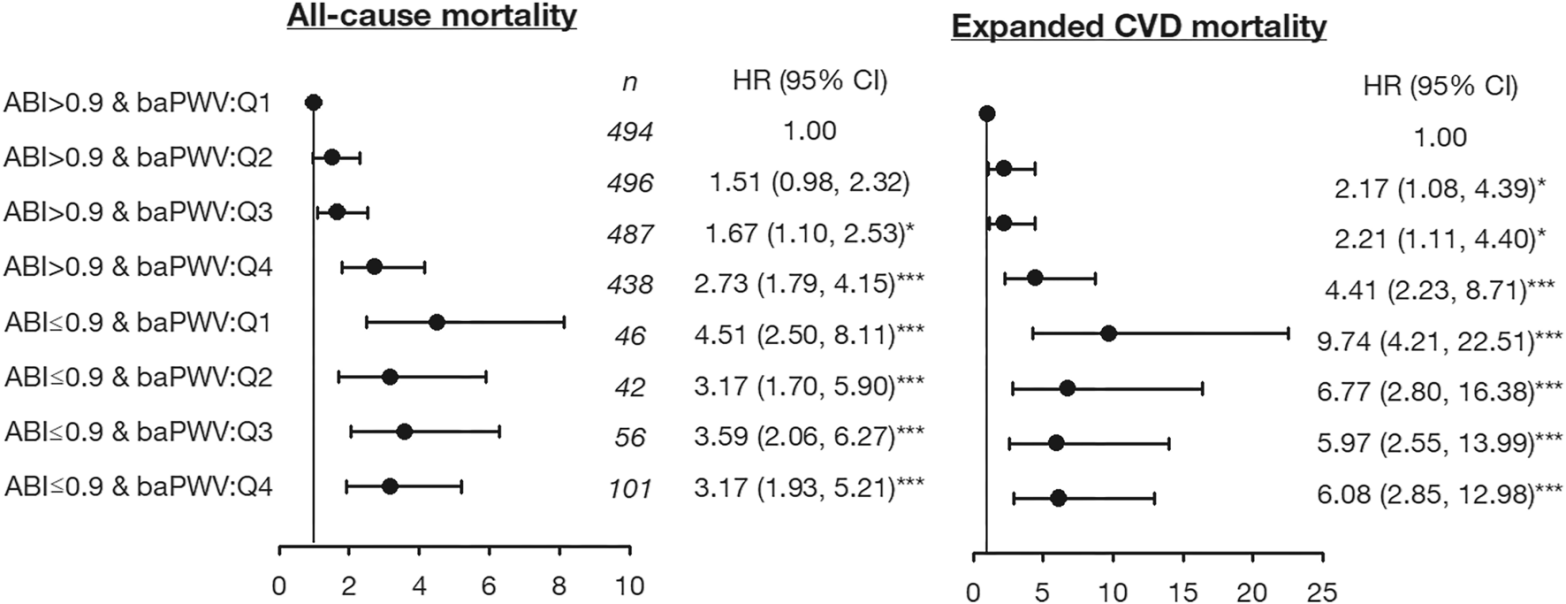
Joint relationship of ABI and baPWV on death incidence.

Figure 2 shows the multivariable-adjusted restricted cubic spline plots of hazard ratios of all-cause and expanded CVD mortality for ABI and baPWV. Non-linear associations were found between ABI plus baPWV and all-cause and expand CVD mortality. Significant interactions were observed between ABI and baPWV in relation to all-cause (χ^2^ with 3 degree freedom [df] for likelihood ratio test = 17.064, *P* for interaction < 0.001) and expanded CVD mortality (χ^2^ with 3 df for likelihood ratio test = 18.173, *P* for interaction < 0.001). baPWV showed an increasing effect under a normal ABI status but a downward trend under an abnormal ABI status for all-cause and expand CVD mortality. Figure 3 shows the risks of all-cause and expanded CVD mortality as determined from the joint effect analysis (with a reference group defined by ABI > 0.90 and baPWV < 1515 cm/s). The adjusted HRs of abnormal ABI with the lst, 2nd, 3rd, and 4th quartiles of baPWV were 4.51 (2.50–8.11), 3.17 (1.70–5.90), 3.59 (2.60–6.27), and 3.17 (1.93–5.21) for all-cause mortality, respectively, and 9.74 (4.21–22.51), 6.77 (2.80–16.38), 5.97 (2.55–13.99), and 6.08 (2.85–12.98) for expand CVD mortality, respectively.

## Discussion

This study identified that after the adjustment for potential confounding factors, an abnormal ABI or high baPWV was related to an increased risk of all-cause and expanded CVD mortality in patients with T2DM at the average follow-up of 8.4 years. The patients with abnormal ABI alone, high baPWV alone, or abnormal ABI plus high baPWV conferred a 1.67-, 1.63-, and 3.17-fold increased risk of all-cause mortality, respectively, and a 2.21-, 1.75-, and 6.08-fold increased risk of expanded CVD mortality, respectively. Significant interactions existed between ABI plus baPWV and all-cause and expanded CVD mortality. baPWV showed an increasing effect under a normal ABI status but a downward trend under an abnormal ABI status for all-cause and expand CVD mortality. ROC results revealed that the combination of ABI and baPWV may improve risk classification in predicting all-cause and expanded CVD mortality in patients with T2DM.

ABI and baPWV are simple, non-invasive, economical, and realistic measures that have become increasingly important in predicting mortality^28–46^. Most studies exploring the associations of ABI and/or baPWV with mortality in T2DM population^28–46^ only considered ABI alone^28–42^. Only two works considered baPWV alone^43,44^, and another two simultaneously considered ABI and baPWV^45,46^. As a consequence, the findings on the association between ABI and mortality were inconsistent^28–42^. Significant associations were observed in some studies^28–40^ but not in others^41,42^. Two prior studies conducted in patients with DM did not detect the predictive capacity of abnormal ABI for mortality, and these non-significant findings can be explained by their small sample sizes^41,42^. Most previous works had relatively small sample sizes (8 studies with *n* < 1000), which limit their ability to detect the effect of ABI on mortality. Although previous studies found that baPWV is an independent predictor for mortality in patients with DM, they cannot rule out the potential confounding effects of lifestyle behaviors (alcohol consumption and physical activity) and type of diabetes medication that were not considered in the multivariate analysis^43,44^. In addition, no prior study has been conducted among Chinese individuals with T2DM. Only two investigations had considered ABI plus baPWV to predict risks in patients with T2DM, i.e., determining the independent effects of ABI and baPWV^45,46^. One found that baPWV, but not ABI, could predict all-cause mortality in patients with diabetes after lower extremity amputation^45^, and the other showed that the combination of abnormal ABI and high baPWV have a stronger association with mortality than normal ABI plus low baPWV in patients with diabetes^46^. The latter work is consistent with the present findings. However, both studies had limitations of short follow-up period (< 6 years) and small sample size (*n* < 500). Our research reported that among the 2,160 enrolled patients, those satisfying both conditions (ABI ≤ 0.9 and 4th baPWV quartile) had a threefold risk of all-cause mortality and sixfold risk of expanded CVD mortality at the mean follow-up of 8.4 years.

Our study showed that mortality was significantly negatively associated with ABI and significantly positively associated with baPWV. Adding ABI and baPWV to baseline characteristics can improve the risk predictive ability for all-cause and expanded CVD mortality. Meanwhile, the significant interactions between ABI and baPWV revealed that their effects are antagonistic. The effect of baPWV slightly decreased in the patients with abnormal ABI but increased in the individuals with normal ABI. However, the interaction between ABI and baPWV has not been previously assessed^45,46^. Potential mechanisms involved in the associations between ABI/baPWV and mortality include oxidative stress, insulin resistance, inflammation, endothelial dysfunction and damage, functional and/or morphological changes in the blood vessel wall, or hemodynamic alterations with arterial stiffness^39,47,48^. The antagonistic interactions between ABI and baPWV may be due to their competing risks, that is, any abnormality on ABI or baPWV leads to unfavorable conditions for the other factor and induces its effect. Future bench research must further clarify the role and interplay of these underlying biological mechanisms of ABI and baPWV. Our study provided clinical information on the prognostication of mortality risk and suggested the use of baPWV and ABI in clinical practice to predict mortality in patients with T2DM.

This study found the combining ABI and baPWV facilitates predictive power for all-cause and expanded CVD mortality. In assessing the strength of association between the outcomes and combination of ABI and baPWV, it shows that the magnitude of association between baPWV and outcomes increases as the baPWV level increases in persons with normal ABI whereas the magnitude of association decreases as the baPWV level increases in persons with low ABI, i.e., low ABI reduce the magnitude of association between baPWV and outcomes. One possible explanation for this phenomenon is the pseudo-underestimation phenomenon of baPWV due to the existence of a significant physical obstacle in the measuring pathway. According to an article reviewing 23 studies exploring baPWV predictive ability on all-cause or cardiovascular diseases^49^, baPWV was a prognostic significance factor in general population, person with diabetes, haemodialysis, acute coronary syndrome, congestive heart failure, hypertension, etc., but no significant association was observed in diabetes if subjects with PAD or low ABI (< 0.9) were not excluded, which can be explained by our findings of the joint effect analysis. It was suggested that the combination use of baPWV and ABI should be practiced in studies to screen patients with suspect PAD through ABI measurement. On the contrary, we found significant association between baPWV and outcomes in persons with low ABI with the lowest quartile of baPWV exhibiting the greatest magnitude of association for all-cause and CVD mortality. A similar trend of association has been reported by a study conducted in heart failure patients with preserved ejection fraction but no peripheral artery disease (PAD) and they found the lowest baPWV level was associated with total cardiovascular events and heart failure-related events with the greatest magnitude of HR, and the results were similar to those with PAD^50^. Although we didn’t have plausible mechanisms to explain these phenomenon, this line of research questions should be explored in future studies because more data are needed to provide evidence on whether simultaneous measurement of ABI and baPWV could further stratify persons with various degrees of vascular damage.

## Strengths and limitations

The major strengths of our study included the longitudinal hospital-based cohort design with regularly standardized collection of clinical and laboratory data in the same clinical laboratory and the sufficiently high quality of definitions for death status and causes to be included in the national death registration data reported to the Taiwan Ministry of Health and Welfare. Our study has two limitations. One is that all patients were recruited from a single hospital central under the case management program, thus possibly limiting the generalization of the results due to variations in healthcare conditions. Nevertheless, the findings are generalizable to the populations with similar characteristics and under similar medical care. In addition, analysis on study subjects under the same clinical setting adds value to the present study for enhanced follow-up rates. The other limitation is that baPWV may not be accurately measured in patients with arteriosclerosis obliterans, aortic valve disease, arterial calcification in the lower limb, etc.^4^, resulting in the possibility of pseudo-underestimation phenomenon of baPWV. This phenomenon may occur due to the existence of a significant physical obstacle in the measuring pathway. In the present study, we cannot exclude those patients with diseases that may result in the pseudo-underestimation phenomenon due to lack of clinical information. If there exists such a misclassification error, persons with true elevated baPWV values might be misclassified as low baPWV level, which result in under-estimation of the effect of abnormal baPWV, indicating this kind of error results in the effect toward the null, a lesser threat to validity. Further studies are needed to pay attention to patients who have sever disease while using ABI and baPWV as predictors of clinical outcomes.

## Conclusion

This study showed that ABI and baPWV were independently and jointly associated with all-cause and expanded CVD mortality in patients with T2DM. ABI plus baPWV improved the prediction of all-cause and expanded CVD mortality in patients with T2DM compared with ABI or baPWV alone. Significant antagonistic interactions were also found between ABI and baPWV. In conclusion, ABI and baPWV measurement is a useful method to predict mortality risk and may be incorporated into daily medical practices to individualize risk assessment and guide clinical decisions.

## Supplementary Information

Supplementary Figure S1.

## Data Availability

The datasets generated and/or analyzed during the current study are not publicly available due to the policy declared by National Health Insurance in Taiwan but are available from the corresponding author on reasonable request.

## Abbreviations

DM: Diabetes mellitus
PAD: Peripheral artery disease
ABI: Ankle-brachial index
PWV: Pulse wave velocity
baPWV: Brachial-ankle pulse wave velocity
AUC: Area under receiver operating characteristic curve
T2DM: Type 2 diabetes mellitus
DCMP: Diabetes Care Management Program
CMUH: China Medical University Hospital
ICD-9-CM: International Classification of Disease, 9th Revision, Clinical Modification
FPG: Fasting plasma glucose
HbA1c: Hemoglobin A1c
HDL-C: High density lipoprotein-cholesterol
LDL-C: Low density lipoprotein-cholesterol
TG: Triglycerides
TC: Total cholesterol
BMI: Body mass index
BP: Blood pressure
CVs: Coefficient of variations
HPLC: High-performance liquid chromatography
CVD: Cardiovascular disease
ICD-10-CM: International Classification of Disease, 10th Revision, Clinical Modification
PPV: Positive predictive value
NPV: Negative predictive value
NRI: Net reclassification improvement
IDI: Integrated discrimination improvement
HR: Hazard ratio
CI: Confidence interval
