# Supplementary material for: Prediction of all-cause and cardiovascular mortality using ankle-brachial index and brachial-ankle pulse wave velocity in patients with type 2 diabetes
Source: Sci Rep. 2022 Jun 30;12:11053. doi: 10.1038/s41598-022-15346-9 (PMC9247028; doi:10.1038/s41598-022-15346-9)
Supplement: Supplementary file 1 — Supplementary Figure S1. [file 41598_2022_15346_MOESM1_ESM.docx]

A total of 18,373 adults were enrolled in the Diabetes Care Management Program

A total of 2,160 participants were included for analysis.

A total of 2,390 subjects were eligible.

Exclusion criteria:

Type 1 diabetes or gestational diabetes (*n* = 448)

Age < 30 years (*n* = 504)

Without ABI or baPWV information (*n* = 15,031)

Enrollment period for NDCMP less than 1 years (*n* =122)

Without sociodemographic factors, lifestyle behaviors, diabetes-related factor, complications, medication use and biomarkers (*n* = 108)

**Supplemental Fig S1.** The flowchart of recruitment procedure for study subjects
